# A novel method for extraction of high purity and high production *Phytophthora sojae* oospores

**DOI:** 10.1186/s13007-024-01199-y

**Published:** 2024-05-16

**Authors:** Xiaomeng Chu, Ziyi Yin, Pengjie Yue, Xinyu Wang, Yue Yang, Jiayi Sun, Ziying Kong, Jian Ren, Xiaohan Liu, Chongchong Lu, Haipeng Zhao, Yang Li, Xinhua Ding

**Affiliations:** College of Plant Protection, Shandong Agricultural University, Tai, Shandong 271018 China

**Keywords:** *Phytophthora sojae*, Oospores, Extraction method, Biological extraction

## Abstract

**Background:**

*Phytophthora sojae*, a soil-borne oomycete pathogen, has been a yield limiting factor for more than 60 years on soybean. The resurgence of *P. sojae *(*Phytophthora sojae*) is primarily ascribed to the durable oospores found in soil and remnants of the disease. *P. sojae* is capable of infesting at any growth periods of the soybean, and the succeed infestation of *P. sojae* is predominantly attributed to long-lived oospores present in soil. Comprehending the molecular mechanisms that drive oospores formation and their significance in infestation is the key for effective management of the disease. However, the existing challenges in isolating and extracting significant quantities of oospores pose limitations in investigating the sexual reproductive stages of *P. sojae*.

**Results:**

The study focused on optimizing and refining the culture conditions and extraction process of *P. sojae*, resulting in establishment of an efficient and the dependable method for extraction. Novel optimized approach was yielded greater quantities of high-purity *P. sojae* oospores than traditional methods. The novel approach exceeds the traditional approaches with respect to viability, survival ability, germination rates of new oospores and the pathogenicity of oospores in potting experiments.

**Conclusion:**

The proposed method for extracting *P. sojae* oospores efficiently yielded a substantial quantity of highly pure, viable, and pathogenic oospores. The enhancements in oospores extraction techniques will promote the research on the sexual reproductive mechanisms of *P. sojae* and lead to the creation of innovative and effective approaches for managing oomycete diseases.

## Background

Soybean root rot, also known as PRR (Phytophthora root rot), is a pervasive and destructive soil-borne disease posing a substantial threat to soybean production worldwide [1–3]. Soybean root rot primarily affects the roots and stems of soybean plants and is capable of infesting at any stage of soybean production [4]. Infected plants’ roots are characterized by a distinct blackened and rotted appearance, featuring stunted root systems and sparse rhizomes. In severe cases, the plants show wilting symptoms and eventually succumb to the disease. Soybean root rot has been shown to cause a substantial decrease in soybean yield, varying between 10–40% [5]. An extreme case cause 100% crop loss [6], leading to significant economic impact on soybean-producing regions globally, with an estimated annual yield loss of $1–2 billion [7]. Additionally, the disease adversely affects the oil content of soybeans, decreasing the overall quality of the crop [6].

The occurrence and damage of Soybean root rot are facilitated by various factors viz soil with a high bacterial count, rainfall, temperature and humidity as environmental factors. Lack of proper seed treatment and the cultivation of susceptible varieties [8] as cultural factors. Various strategies were implemented to manage the disease which includes cultural, physical, biological, and chemicals [9–11]. However, existing control measures are inefficient and susceptible for cross-contamination between fields due to the ability of the pathogen to persist in the soil for up to a decade and ability to get transmitted through the soil [4]. Thus, it is essential to acquire an in-depth understanding of the genetic basis of soybean root rot to devise management strategies that are environmentally sustainable and effective. Four pathogenic bacteria, namely *P. sojae*, *Fusarium* spp., *Pythium* spp., and *Rhizoctonia solani*, have been identified as the causative agents of soybean root rot. Among these, *P. sojae* stands out as highly pathogenic [11].

*P. sojae* is a multicellular, septate, nuclear filamentous pathogen that specifically parasitizes plants belonging to the legume family, including soybean [1, 12]. *P. sojae*, first identified in India and USA, And is the earliest oomycete pathogens to have its genome sequenced in 1948 [13]. This has positioned it as a model organism for oomycete research, thanks to the abundance of genomic data and the availability of advanced gene editing technique [14–17]. *P. sojae* has evolved a varied lifestyle and is capable of both sexual and asexual reproduction. In its asexual reproductive cycle, *P. sojae* produces zoosporangia and zoospores. Zoospores, equipped with two flagella [18], can swim in water or transition into resting spores. Notably, these zoospores exhibit a strong attraction to isoflavonoids soy glycosides released by soybean roots [1, 19]. Upon detecting their host, they employ germ tubes to infiltrate the roots and commence infection [20]. In the sexual reproduction phase, *P. sojae* forms oospores [21]. Distinguished by their thick walls and is the unique characteristics of oospores that distinguish them from other spores [22]. This characteristic allows them to endure harsh conditions and survive for prolonged periods in the soil [23]. . In contrast to short-lived spores, which struggle to persist outside the host, oospores demonstrate exceptional resilience, withstanding freezing temperatures, fungicide applications and maintain viability for multiple years, presenting a persistent threat in agricultural environments [24].

Oospores are vital in the infestation cycle of oomycetes, serving as the most enduring overwintering structures and the primary inoculum source for reinfecting host plants in successive growing seasons. In both homothallic and heterothallic species, male and female gametangia develop and merge to produce oospores. The oospores formation process includes several critical stages, including host plant infection, nuclear fusion, nuclear division, oospores wall formation, and maturation [25]. A notable feature of oospores is their extended survival in soil and plant debris attributed to their thick cell walls and innate dormancy [23]. These spores can endure a broad temperature spectrum from freezing to 40 °C. This adaptability is especially significant in homothallic species which are self-fertilizing and do not require mating partners for oospores production. The combination of thick cell walls, endogenous dormancy and temperature tolerance enables oospores to persist under diverse environmental conditions ensuring its continuous survival and propagation.

The complexity and laboriousness are the major hurdle in high purity oospores extraction process. Several traditional methods including high-speed homogenization followed by filtration through sieves, coarse cotton cloths or filters, as well as direct wash concentration, have been employed for *P. sojae* oospores extraction [26–29]. Moreover, current extraction methods for other oomycete oospores (*spinach downy mildew* and *P. capsici*) are limited to filtration using tools such as cotton cloths [30, 31], which is conceivably inefficient. However, these methods present certain limitations. Direct washing frequently leads to excessive agar in the oospores suspension, whereas conventional filtration techniques are time-intensive and may cause considerable oospores loss. Additionally, the challenge of mycelial contamination removal in traditional oospores extraction is often overlooked. In some cases, materials used in oospores-related studies have been incorrectly identified as mycelial materials [32, 33] leading to inconsistencies in experimental materials and potentially impacting the reliability of experimental outcome. All these underscores the importance of advancing these extraction techniques, and developing innovative methods that reduce agar contamination and enhance oospores recovery will enable more in-depth studies into the development and functions of oospores in oomycetes.

## Materials and methods

### Strain cultivation

In this study, we used the homologous *P. sojae* strain P6497, which we routinely maintained on 10% V8 solid medium (100 mL V8 juice from Campbell Soup Company, 1.4 g CaCO_3_, 15 g agar, distilled water to 1 L) in dark at 25 °C. To investigate the effect of agar content on the growth of *P. sojae*, V8 solid media supplemented with agar at concentrations of 3.75 g/L, 7.50 g/L, 11.25 g/L, and 15.00 g/L were prepared in addition to conventional 10% V8 media and then inoculated *P. sojae* on each type of medium and incubated it at 25 ℃ in dark for 7 days.

### Oospores count

After 7 days of incubation, the cultured that we obtained a 0.5 cm×0.5 cm block from a location 2 cm away from the mushroom cake using a scalpel. The sample was subsequently placed on a slide, after which five separate fields of view were selected under a microscope (BM1800) at 4X magnification to calculate the average number of oospores. The plates were first blended with 200 mL of sterile water at low speed for 2 min, creating a homogenized mixture of oospores and culture medium. The homogenate was transferred to a sterilized conical flask and stirred at 37 °C for 20 min until it clarified, facilitating the stratification of oospores and the culture medium. The oospores were subsequently transferred to 50 mL centrifuge tubes and centrifuged at room temperature for 10 min at 9,000 rpm. After removing the supernatant, the oospores sediment was visible at the bottom of centrifuge tube. To eliminate excess medium, the upper layer was carefully removed with a medicine spoon, and 20 mL of deionized water was added. This process was repeated thrice to yield an oospores suspension containing mycelia. Further, lysing enzyme at a concentration of 2 mg/mL was added to the suspension. The suspension mixed with lysing enzyme (Yuanye S10107), was incubated for 4 h at 20 ℃ and 60 rpm, aids to remove mycelia and resulting in a purified oospores suspension. For analysis, 30 µL of this oospores suspension was used to create a film. The oospores count was then determined under a BM1800 microscope (4×magnification), and the procedure was repeated three times to calculate the average oospores count. Additionally, the morphology of the oospores was examined under a BM1800 microscope (40×magnification).

### Viability assays

For staining the oospores suspension, 5 mg/mL MTT dye solution was prepared by dissolving 0.5 g MTT from Solarbio, in 100 mL PBS. This solution was then filtered through a 0.22 μm membrane and stored at 4 ℃ in dark. To proceed with the staining, 800 µL of the oospores suspension was combined with 200 µL of MTT dye in a 2 mL tube. This mixture was incubated at 37 ℃ for 24 h. Following incubation, the color of the spores were examined: those stained rose or purple were considered positive for viability, while spores that were not stained or stained black were counted as negative. We calculated the survival rate of the oospores using the following formula: number of purplish-red oospores / total number of oospores*100%. In the beginning we prepared WA (20 g agar, 1000 mL deionized water) culture plates supplemented with Rifampicin, Ampicillin, and Pentachloronitrobenzene. The oospores suspension was treated with 0.1% KMnO_4_ for 10 min, then washed five times with PBS. Subsequently, 800 µL of this treated suspension was added to a culture plate. An inactivated applicator was used to spread the suspension evenly across the plate which was then dried and inverted in lighted environment at room temperature. Oospores germination was monitored every 24 h, with germination typically occurring around 48 h. To calculate the rate of oospores germination, we used the following formula: number of germinated oospores / total number of oospores*100%.

### Infection assays

A total of 1 × 10^4^ oospores were incorporated into 2 kg of soil then evenly divided into several pots. Each pot was planted with 10 susceptible soybean seeds (variety ZH13). Disease incidence was observed at 7 days of post inoculation. This procedure was replicated three times for each treatment group to maintain consistency. The random assignment of pots, uniform watering, and cultivation practices ensured the randomness of the experiment. Moreover, to confirm the reliability of the findings, this experiment was conducted independently for three times. The disease incidence was calculated using the formula: 1 - (number of germinated soybean plants / total number of soybean plants*100%).

## Results

### Optimization of culture conditions

**Fig. 1:**
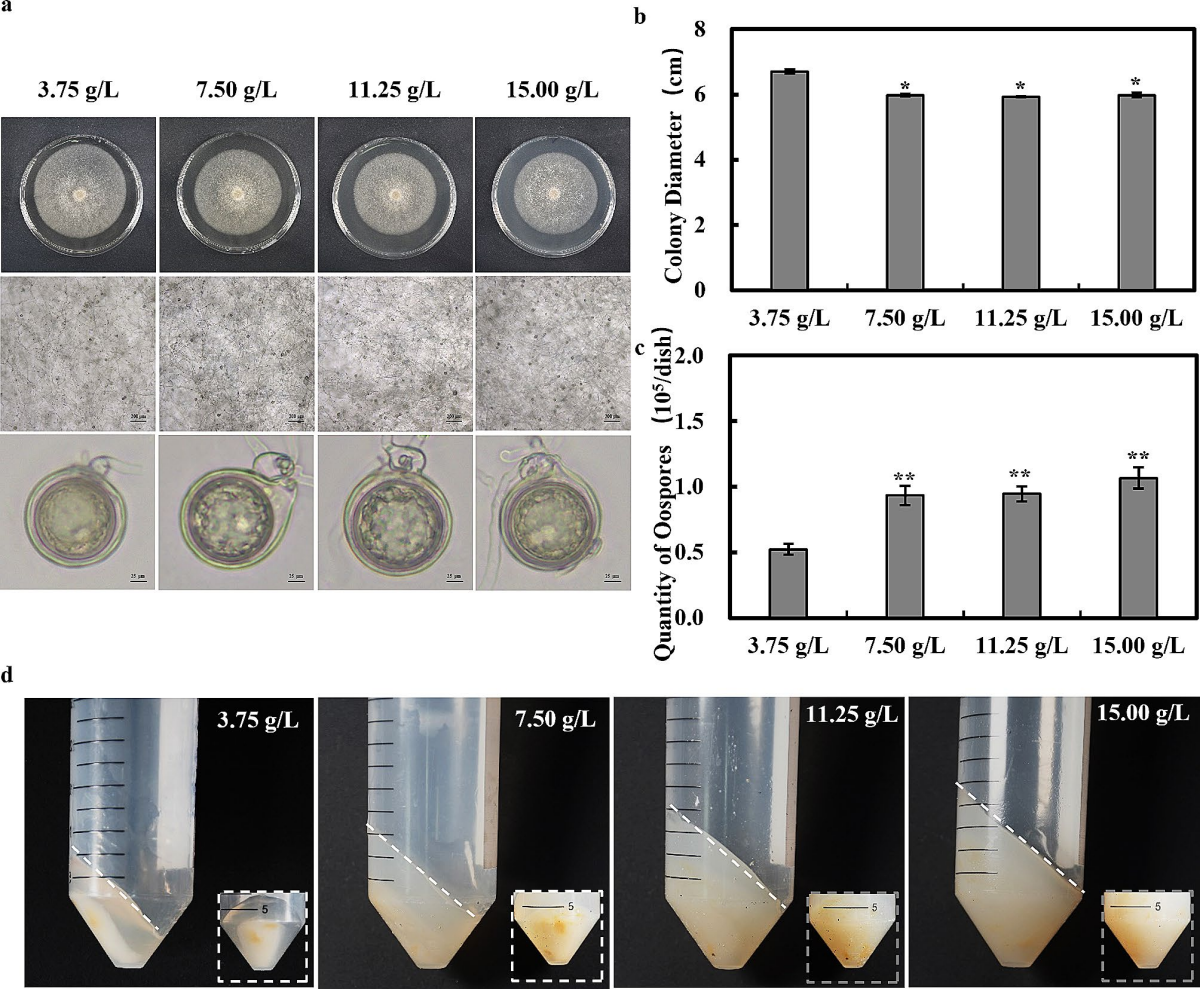
Effect of different agar content media on *phytophthora sojae* oospores production. (**a**-**c**) Evaluation of colony diameter, oospores production (4X), and oospores morphology (40X) of *Phytophthora sojae* after 7 days of growth on media with varying agar contents. (**d**) Assessment of agar residues of *Phytophthora sojae* during oospores extraction and deposition of oospores at the bottom of centrifuge tubes after 7 days of growth on media with different agar contents. Values presented are the mean ± standard deviation (SD) of three biological replicates. *P ≤ 0.01 (unpaired t-test)

The traditional method of oospores extraction by growing *Phytophthora sojae* on 10% V8 solid medium with an agar content of 15.00 g/L encountered a large amount of agar residue and makes the extraction process difficult. To overcome this issue and to obtain a higher yield of pure oospores, we conducted experiments to adjust the agar content of 10% V8 solid medium. The results revealed that reducing the agar content to 7.50 g/L led to a substantial decrease in agar residue during the oospores extraction process. Additionally, a higher number of oospores were deposited at the bottom of the centrifuge tubes (Fig. 1d). Importantly, this adjustment had no notable impact on the mycelium’s growth rate, oospores morphology, or oospores production (Fig. 1a-c). However, further reduction of the agar content to 3.75 g/L had a significant adverse effect on both the mycelial growth rate and oospores production. Therefore, 10% solid V8 medium with an agar content of 7.50 g/L was the optimal choice for cultivation of *P. sojae*. This optimization ensures the stability of spore production, minimizes residual agar, and improves the purity of oospores extraction.

### Optimization of extraction methods

**Fig. 2:**
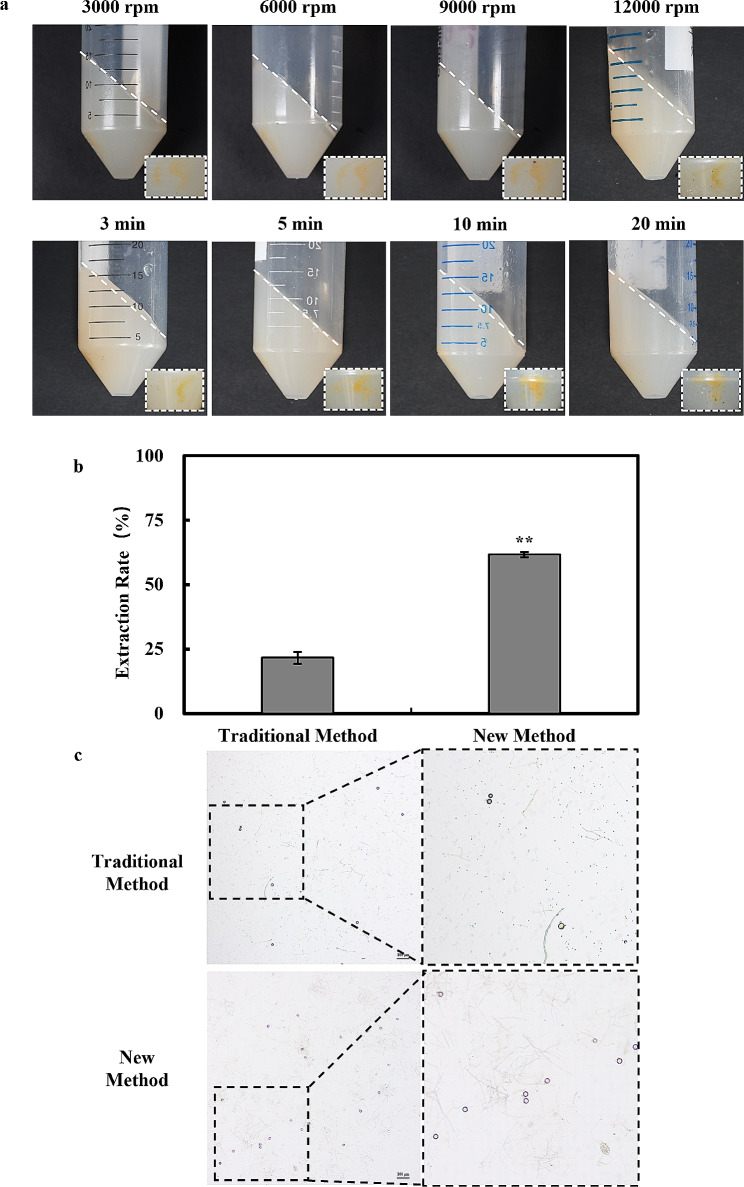
Illustrates a comparison of the extraction rates achieved by the new method and the traditional method for extracting oospores. (**a**) The optimal centrifugal speed was screened by evaluating the amount of agar residue remaining after centrifugation of high-speed homogenized *Phytophthora sojae* plates at different speeds and times, as well as the deposition of oospores at the bottom of the centrifuge tubes. (**b**-**c**) A comparison was made between the number of oospores extracted using the optimized new method (4X) of *Phytophthora sojae* oospores extraction and the extraction rate obtained with the traditional method. Extraction rate = (the number of oospores extracted / the number of oospores after homogenization) * 100%. T Values represent the mean ± standard deviation (SD) of three biological replicates. *P ≤ 0.01 (unpaired t-test)

We observed a low oospores extraction rate using the traditional extraction method, prompting us to optimize and improve the existing approach. Through our experiments, we successfully developed and optimized new extraction method, and the steps are as follows. One plate was well homogenized and stirred at 37 °C until clarified, then centrifuged at room temperature for 10 min to settle the oospores at the bottom of the centrifuge tube. The excess medium was gently removed from the upper layer with a medicated spoon and the process was repeated three times to obtain a suspension of oospores containing mycelium. This new extraction method significantly increased the extraction rate to over 60% in comparison with the traditional extraction method which yielded only 26%. The optimized method resulted in a 2.3-fold increase in oospores extraction rate compared to the traditional method (Fig. 2b-c). Furthermore, we conducted a screening process to determine the optimal centrifugation speed and optimal centrifugation time. Experimental results revealed that the oospores were most effectively deposited into the lower layer of agar when the centrifugation speed was set at 9000 rpm at 10 min (Fig. 2a). Based on these findings, we selected the centrifugation speed of 9000 rpm for subsequent extraction steps.

### Oospores purification

**Fig. 3:**
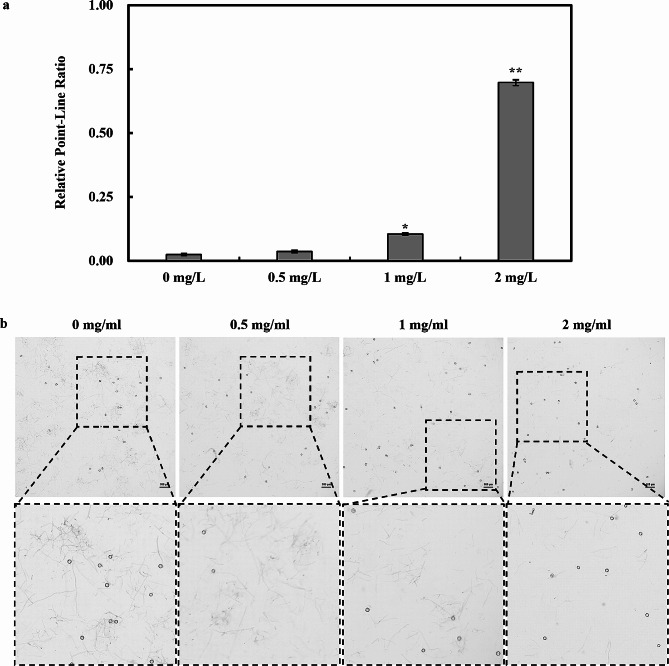
Investigation into the impact of different concentrations of lysing enzyme on the presence of mycelium in oospores suspensions. After enzymatic hydrolysis of oospores suspensions using various concentrations of lysing enzyme (4X), the amount of mycelium in five random fields of view was examined microscopically. Statistical analysis of the relative point-line ratio was then conducted. Relative point-line ratio = the number of oospores / the number of mycelial strips. Values represent the mean ± standard deviation (SD) of three biological replicates. *P ≤ 0.01 (unpaired t-test)

After the successful implementation of the optimized new method, a notable increase in oospores extraction rate was achieved. However, the resultant oospores suspensions contained a substantial amount of mycelium hence leading to reduced oospores purity. To address this issue, a purification process utilizing lysing enzymes was introduced. Experimental findings demonstrated that enzymatic digestion of the oospores suspension agitation for 4 h at room temperature and 60 rpm effectively reduced the presence of mycelium. Notably, the concentration of lysing enzyme played a pivotal role in this process, as an increase in enzyme concentration corresponded to a significant decrease in mycelium observed under microscopic examination. At a lysing enzyme concentration of 2 mg/mL, the mycelium content in the oospores suspension was substantially reduced, and the Relative Point-Line Ratio increased from approximately 0.02 to about 0.7 (Fig. 3), indicating a remarkable enhancement in oospores purity. Consequently, within the optimized new method, the chosen purification approach involved enzymatic digestion using 2 mg/mL of lysing enzyme at room temperature with agitation at 60 rpm for 4 h.

### Oospores viability verification

**Fig. 4:**
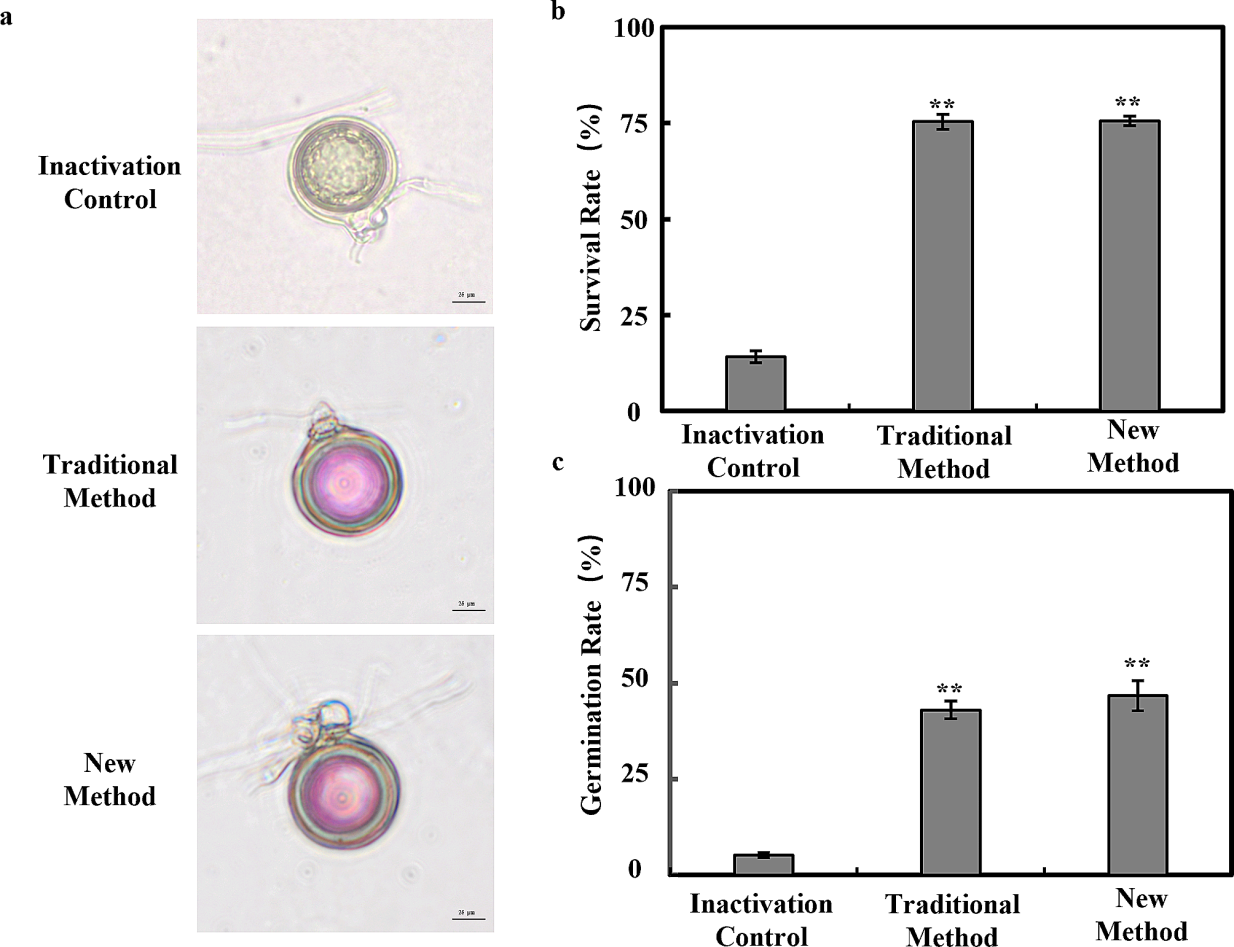
Viability comparison of oospores extracted by new and traditional methods in *phytophthora sojae*. (**a**) Morphology of viable oospores after MTT treatment. (**b**) Comparison of oospores viability extracted using the new method and traditional method in *Phytophthora sojae* oospores extraction, following MTT treatment. Viability = the number of purplish-red oospores / the total number of oospores * 100%. (**c**) Comparison of the germination rate of *Phytophthora sojae* oospores extracted by the new method and the traditional method, assessed by plate coating with KMnO_4_ treatment. Germination rate = the number of germinated oospores / the total number of oospores * 100%. Values represent the mean ± standard deviation (SD) of three biological replicates. *P ≤ 0.01 (unpaired t-test)

Spore viability was assessed using the thiazolyl blue tetrazolium bromide (MTT) staining technique. Viable oospores appeared purple, while non-viable oospores were either black or unstained. The MTT assay results revealed that the viability of oospores extracted using the new method did not significantly differ from that of the traditional method, with both methods yielding approximately 75% viability (Fig. 4a-b). Additionally, the oospores germination rate serves as an important indicator of spore viability. The results demonstrated that the germination rate of oospores in the new method-extracted oospores suspension was not significantly different from that of the traditional method, averaging around 50% (Fig. 4c). In conclusion, the optimized new method yielded similar oospores with survival and germination rates comparable to the traditional method, indicating that oospores viability remained unaffected.

### Oospores pathogenicity verification

Soybean plants were cultivated in nutrient soil mixed with a suspension of *P. sojae* oospores. Experimental results revealed that the disease incidence in soybean plants grown in nutrient soil mixed with oospores suspension extracted using the new method was not significantly different from those grown in nutrient soil mixed with oospores suspension extracted using the traditional method. The disease incidence in both cases was approximately 50%. These findings indicate that there is no significant difference in the pathogenicity of the oospores extracted using the optimized method compared to the traditional method.

## Discussion

Comprehending *P. sojae* sexual reproductive mechanism is crucial for oomycete diseases management. However, existing imperfections of the traditional methods in oospores isolation and extraction resulting in poor harvests of high purity oospores severely restricts this reproductive mechanism investigation. In this study, an improved method for extracting *P. sojae* oospores is presented through the optimization of culture conditions and extraction methods.

Oospores extraction yield is affected by multiple factors, one of them is culture conditions. Traditional oospores extraction methods yield impure oospores with residual agar, posing difficulties for subsequent experiments. In addressing this challenge, the effect of media with varying agar contents on oospores production was investigated. It was determined that the agar concentration in the solid medium is inversely proportional to the residual agar level and *P. sojae* oospores production, and reducing the agar content to 7.50 g/L did not adversely affect mycelial growth rate, oospores morphology, or oospores production which was optimal for subsequent culture experiments. However, inadequate agar content hampers oospores production. Similarly, Smart *et al*. proved that liquid media impede oospores production [34], which congruent with our observations regarding the impact of varying agar contents on *P. sojae* oospores production. Agars are widely used in biological and microbiological experiments as common solidifying agents and medium components, but agar itself can influence the pH of the medium, usually rendering it weakly acidic. Bhagyashali V Hudge *et al.* suggested that pH affects *Pythium ultimum* oospores production [35], we hypothesized that *P. sojae* oospores production could also be affected by pH. Due to the absence of corroborating evidence, the conclusions remain speculative, and we aim to conduct further experiments to elucidate this matter.

Oospores production can be improved by adjusting other culture mediums to increase oospores production. Smart *et al.* demonstrated that a solid medium composed of V8 juice, tomato juice, and oatmeal effectively promoted oospores production in type A2 strains [34]. Additionally, they identified that porous carbonate membranes and diffusible substances enriched in V8 juice also stimulated oospores formation. In the present study, greater quantities of oospores are yielded in V8 10% solid medium than the others. In consequence, a solid V8 medium supplemented with 7.50 g/L agar is the most suitable for *P. sojae* cultivation. Moreover, alterations in environmental factors such as temperature, humidity, and light may also exert an influence on oospores production. Cohen *et al.* demonstrated that the optimal temperature for the sexual reproduction of *Phytophthora infestans* ranged from 8 to 15 °C, although oospores could still be generated at 23 °C [36]. According to the results of previous studies, incubating *S. physalifolium* at an optimum temperature and 100% relative humidity promotes oospores formation [37]. Therefore, the extraction method can be optimized in more detail by varying the various culture conditions to increase the amount of oospores production and thus the number of oospores extracted at the end, and we will follow up this part of the optimization in subsequent studies.

In this study, we have tested and compared to form a method of oospores extraction with high extraction rate. Upon testing and comparison, the optimized method demonstrates a 2.3-fold increase in the extraction rate compared to the traditional method, reaching approximately 60%. Incubating the plates for approximately 7 days yielded approximately 60,000–70,000 oospores (Fig. 2). Currently, the traditional method of oospores extraction usually uses homogenization followed by sieving to extract the oospores, but after repeating the traditional extraction method, we found that many oospores were lost after sieving and the oospores were hardly separated from the agar. Therefore, we separated the oospores and agar as much as possible by stirring at 37 °C and then centrifuged the agar so that the majority of oospores were deposited in the lower layer of the agar and then removed the agar by using the spatula. Stirring helps to accelerate the contact and diffusion of the small particles of agar with the warm liquid and facilitates the dissolution process. Over time, the small particles of agar will gradually dissolve into a liquid state and be evenly distributed in the liquid. It has been shown that oospores exposed to soil at 36 °C for 12 months had a survival rate of 22% and a germination rate of 19%. Although survival and germination rates were low, these oospores can still be used as an initial source of inoculum [38]. In this study we only placed the oospores at 37 °C for 20 min, and its survival ability, viability and germination didn’t harm in the above study (Fig. 4), and we speculate that the reason for this is the thick-walled oospores can tolerate higher temperatures for a short period of time.

**Fig. 5:**
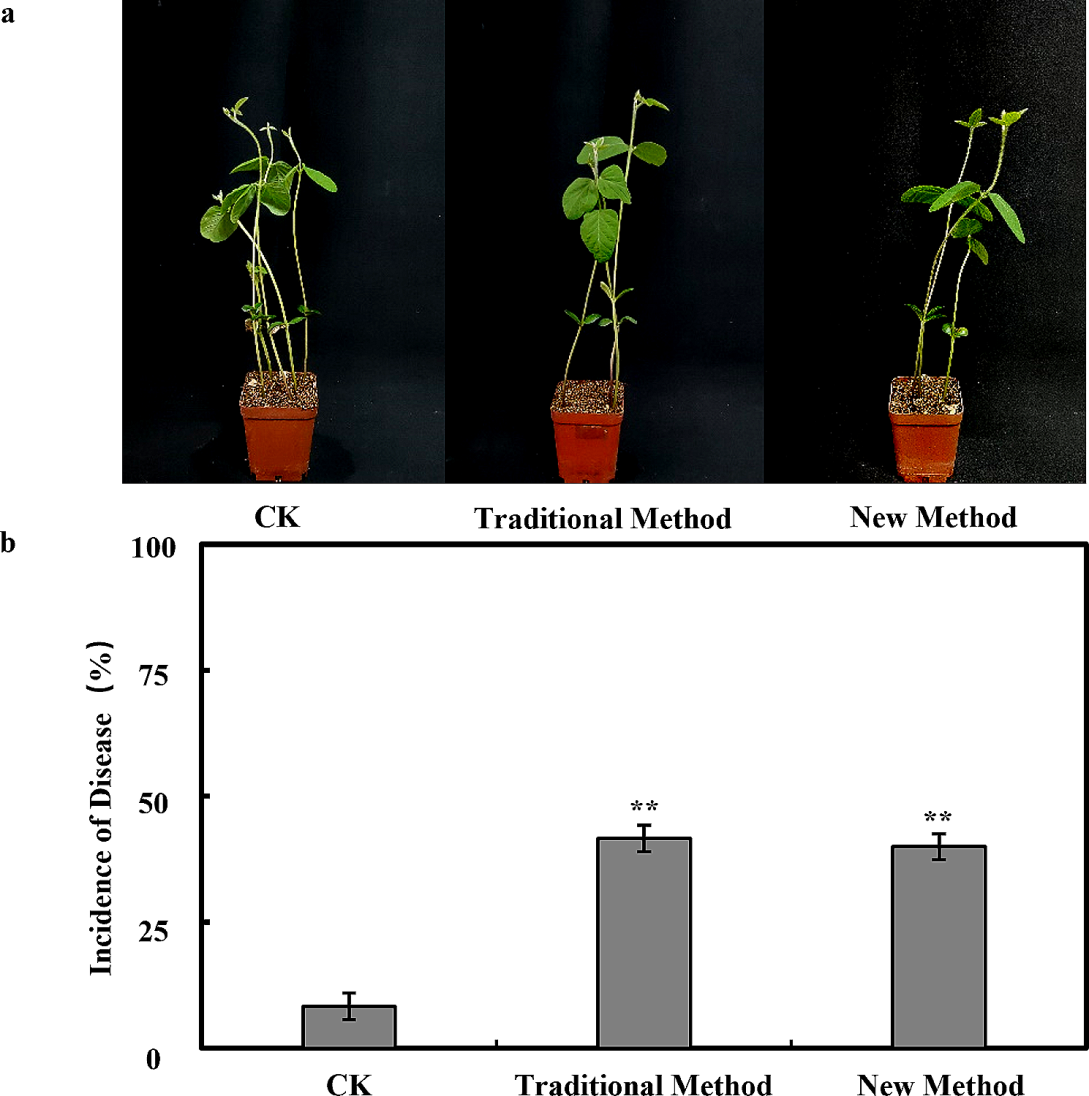
Comparison of pathogenicity of oospores extracted by new and traditional methods in *phytophthora sojae*. Soybean plants were cultivated in nutrient soil mixed with oospores suspensions extracted using the new and traditional methods of *Phytophthora sojae* extraction. The disease incidence was observed after 7 days. Disease incidence was calculated using the formula: 1 - (germinated soybean plants / total number of soybean plants) * 100%. Values represent the mean ± standard deviation (SD) of three biological replicates. *P ≤ 0.01 (unpaired t-test)

The purification process of oospores has important implications for improving the oospores extraction rate. Traditional method obtains low purity of the oospores due to the residue of the mycelia in suspensions. Here, it was proved that the concentration of the lysing enzyme was vital in oospores purification. According to the microscopic examination results, higher enzyme concentration resulted in marked reduction in mycelia, and the mycelium content in the oospores suspension, while the relative point-line ratio was significantly increased with 2 mg/mL lysing enzyme digestion for 4 h at room temperature and 60 rpm before purification (Fig. 3). The survival, germination rate and the pathogenicity of purified oospores were not significantly affected (Fig. 4), and the same was proved upon planting soybean seeds in soil mixed with the harvested *P. sojae* oospores, which were extracted via optimized method, where typical wilting and seedling mortality symptoms were observed within five days. (Fig. 5). Typically, for an extraction method, it is of greater importance to shorten the extraction time without any impact to the yield of the extractants. Previous work has shown that the traditional approach is time-consuming and leads to significant oospores loss along with agar during filtration [27]. For instance, filtering numerous plates alone requires 5 h in the traditional method, and when combined with other steps like homogenization and purification, it takes at least one day to complete the extraction process. Additionally, if the incubation period is extended to increase oospores yield, an additional month is needed to prepare experimental material. In contrast, the new oospores extraction method developed in this study significantly reduces extraction time and plate usage. It takes approximately 4.5 h to complete all extraction and purification steps. Therefore, the newly developed method reduces the experiment duration and maximizes oospores extraction while preventing mycelial contamination.

In summary, an efficient and dependable method for *P. sojae* oospores extraction has been proposed through the optimization of cultural conditions and extraction procedures. This method yields high-purity and high-yield *P. sojae* oospores with exceptional viability and pathogenicity. Furthermore, it is crucial to recognize the potential limitations and scope of application associated with this method. This study specifically concentrated on oospores extraction from the well-known oomycete *P. sojae*. Nevertheless, *P. sojae* serves as a biological model for oomycete research and exhibits morphological and structural resemblances to oospores of other oomycetes along with similarities in reproductive mode and function. We postulate that this method may find utility in optimizing and improving oospores extraction in other oomycetes. Specific investigations in this area are needed for further validation. This finding underscores the importance of our newly developed method, which has the potential to furnish dependable experimental materials for the examination of sexual reproduction in oomycetes. Research endeavors should be facilitated, and crucial groundwork should be established for the refinement of oomycete management strategies.

## Conclusion

Through rigorous testing and optimization of conventional *Phytophthora sojae* oospores extraction methods, we have developed a novel and improved procedure for obtaining high-quality oospores. The optimized method involves several steps: firstly, a plate containing 200 mL of sterile water is blended thoroughly for approximately 2 min to achieve a homogenized mixture of oospores and medium. Next, the homogenate is transferred to a sterilized conical flask and stirred at 37 °C for 20 min until it becomes clear, facilitating the stratification of oospores with the medium. The oospores are then carefully collected into 50 mL centrifugal tubes and centrifuged at room temperature (9000 rpm) for 10 min, allowing the separation of oospores from the medium. After discarding the supernatant, the oospores are observed to settle at the bottom of the centrifuge tube. Excess medium in the upper layer is gently removed using a medicine spoon, repeating this process three times to obtain an oospores suspension containing mycelium. Subsequently, the oospores suspension is treated with 2 mg/mL of lysing enzyme and subjected to enzymatic digestion for 4 h at 20 ℃ and 60 rpm, effectively eliminating the mycelium and yielding a purified oospores suspension. This optimized method provides a rapid and easily implementable tool that significantly enhances our understanding of the molecular mechanisms underlying Oomycete infestation of plants and pathogenicity. By consistently generating highly pure and reproducible oospores, this standardized and scalable approach enables large-scale studies pertaining to the sexual reproduction of Oomycetes. Such investigations are crucial for comprehending Oomycete reproductive modes, transmission pathways, and ecological adaptations which ultimately contributing to the development of scientifically sound disease control strategies.

## Data Availability

The datasets used in this study is available from the corresponding author on reasonable request.
